# Genome-wide neonatal epigenetic changes associated with maternal exposure to the COVID-19 pandemic

**DOI:** 10.1186/s12920-023-01707-4

**Published:** 2023-10-30

**Authors:** Kristen Kocher, Surajit Bhattacharya, Nickie Niforatos-Andescavage, Miguel Almalvez, Diedtra Henderson, Eric Vilain, Catherine Limperopoulos, Emmanuèle C. Délot

**Affiliations:** 1 Center for Genetic Medicine Research, Children’s National Research & Innovation Campus, Washington, DC USA; 2 Department of Genomics & Precision Medicine, George Washington University, Washington, DC USA; 3 Developing Brain Institute, Children’s National Hospital, Washington, DC USA; 4 Institute for Clinical and Translational Science, University of California, Irvine, CA USA

**Keywords:** Epigenetics, DNA methylation, SARS-CoV-2, COVID-19 pandemic, Perinatal stress

## Abstract

**Background:**

During gestation, stressors to the fetus, including viral exposure or maternal psychological distress, can fundamentally alter the neonatal epigenome, and may be associated with long-term impaired developmental outcomes. The impact of in utero exposure to the COVID-19 pandemic on the newborn epigenome has yet to be described.

**Methods:**

This study aimed to determine whether there are unique epigenetic signatures in newborns who experienced otherwise healthy pregnancies that occurred during the COVID-19 pandemic (Project RESCUE). The pre-pandemic control and pandemic cohorts (Project RESCUE) included in this study are part of a prospective observational and longitudinal cohort study that evaluates the impact of elevated prenatal maternal stress during the COVID-19 pandemic on early childhood neurodevelopment.

Using buccal swabs collected at birth, differential DNA methylation analysis was performed using the Infinium MethylationEPIC arrays and linear regression analysis. Pathway analysis and gene ontology enrichment were performed on resultant gene lists.

**Results:**

Widespread differential methylation was found between neonates exposed in utero to the pandemic and pre-pandemic neonates. In contrast, there were no apparent epigenetic differences associated with maternal COVID-19 infection during pregnancy. Differential methylation was observed among genomic sites that underpin important neurological pathways that have been previously reported in the literature to be differentially methylated because of prenatal stress, such as *NR3C1*.

**Conclusions:**

The present study reveals potential associations between exposure to the COVID-19 pandemic during pregnancy and subsequent changes in the newborn epigenome. While this finding warrants further investigation, it is a point that should be considered in any study assessing newborn DNA methylation studies obtained during this period, even in otherwise healthy pregnancies.

**Supplementary Information:**

The online version contains supplementary material available at 10.1186/s12920-023-01707-4.

## Introduction

We and others have documented that maternal psychological distress, such as stress, anxiety, and/or depression, during the prenatal period has been strongly correlated with impaired fetal development and long-term neurodevelopmental and cognitive consequences throughout the child’s life [1–9]. Large-scale traumatic events, such as natural disasters, war, genocide, famine, and pandemics, have been studied for their acute impact on pregnancy, maternal mental health, and subsequent neonatal outcomes [1, 7, 8]. The Dutch Hunger Winter (1944–45) and Canadian Ice Storm (1998) are exemplar traumatic events that have been studied for the postnatal consequences associated with maternal distress and trauma [10–16]. These studies have leveraged DNA methylation analysis to uncover mechanisms associated with environmental stress, the impact on maternal mental health, and how differential methylation of the fetal genome may affect developmental outcomes [17–20]. Numerous studies of maternal perinatal and early life stress suggest that there are critical changes to the entire newborn epigenome that may underlie lifelong developmental and neurological outcomes in offspring [3, 7–10, 21–23]. The present study builds on what is known about epigenetic changes detected in cord blood, by assessing epigenetic associations with exposure to an unprecedented global pandemic utilizing buccal swabs collected from neonates in the first month of life [22, 23]. Notably, Sammallahti et al. have highlighted that cord blood shows inconclusive evidence of the association between DNA methylation changes associated with maternal anxiety [23]. It is thus of the utmost importance to expand the study perinatal maternal distress and other exposures on DNA methylation to multiple tissue types and timepoints, such as newborn buccal swabs, as the epigenome is known to vary considerably as a function of time and cell type. The nexus of perinatal maternal stress and epigenomic research is critical to providing a foundation for therapeutic intervention during pregnancy and beyond to improve both maternal and fetal outcomes.

The sudden onset of the COVID-19 pandemic has provided multiple causes of both acute and chronic stress with recession, isolation, reduced access to healthcare, and fear of, or exposure to, the COVID-19 virus. Since March 2020 when the COVID-19 pandemic was deemed a “natural disaster” in the United States by FEMA, the global health burden of disease has climbed to over 400 million confirmed cases and over 6 million deaths, with new variants of the virus emerging frequently [24]. As a result, the COVID-19 pandemic has been correlated with a trend of increased stress, anxiety, and depression among the global population [25], with pregnant women being no exception from the trend [26, 27].

To document the impact of maternal exposure during the COVID-19 pandemic on fetal development, we began recruitment of pregnant women into the RESCUE Project (Reducing Elevated Stress from COVID-19 Exposure Project) research study in June 2020. For the scope of this study, we sought to characterize the neonatal epigenetic landscape in healthy pregnancies delivered before and after the onset of COVID-19, using pre-pandemic newborn controls (CTL cohort) as a direct comparison to Project RESCUE newborns (RES cohort) recruited from the greater Washington DC Metropolitan region. Due to the varying policies during the COVID-19 pandemic along with varying periods of related surges, we sought to reduce potential confounding factors by focusing on a small, controlled regional cohort with participants largely exposed to the same environment and recruitment schema before and during the COVID-19 pandemic [28]. This study provided a unique opportunity to uncover potential signatures of distress during pregnancy on infant DNA methylation and whether these may predict long-term resilience or susceptibility to adverse neurodevelopmental and behavioral outcomes.

## Material and methods

### Maternal-Infant data

Demographic and clinical data were extracted from maternal questionnaires and medical records. Women eligible for this study were recruited from the Washington DC-Metropolitan area (Virginia, Washington DC, Maryland) and met the following criteria: to be over the age of 18 years with a singleton pregnancy of 8 weeks gestation or greater and to be diagnosed with or are exhibiting symptoms of stress, anxiety, or depression, or have no other health risk factors outside of being pregnant during the COVID-19 pandemic. Additionally, newborn infants born to mothers who tested COVID-19 positive during pregnancy were enrolled and participate in all post-natal portions of the study. Exclusionary criteria for all cohorts included: fetuses/newborns with known chromosomal syndromic conditions, women unable to enter the MRI scanner for physical or psychological reasons, or women who have health conditions that make their pregnancy high-risk. Maternal exposure to and fetal gestational age (GA) at time of COVID-19 infection was captured from medical records and test results. The Coronavirus Perinatal Experiences- Impact Survey (COPE-IS) and the COPE Impact Update (COPE-IU) were also completed to measure the experiences of new and expectant mothers in the time of the Coronavirus COVID-19 (SARS-CoV-2) pandemic [29]. Infant gestational age (GA), sex, birthweight, head circumference, and length at birth, as well as maternal age were recorded at the time of visit. Race and ethnicity information was self-reported. These metrics were assessed for statistical differences using a paired t-test and *p*-value ≤ 0.05, as well as z-score, when applicable (Table 1). Maternal mental health assessments included several self-reported surveys; The State-Trait Anxiety Inventory was used to document acute (STAI-S) and chronic (STAI-T) anxiety [30, 31]; Perceived Stress Scale (PSS) to measure stress [32, 33]; and the Impact of Events Scale-Revised [34] to assess for post-traumatic stress disorder (PTSD) symptoms. Scoring was performed and aggregate data was analyzed for “peak,” or highest possible scoring metric, throughout pregnancy and at birth and that “peak” score was used to label the sample with "low", “moderate”, or “high” STAI-S, STAI-T, and PSS (or “yes” or “no” PTSD). (The specific calculations for all maternal mental health surveys can be found in Supplemental Table S1).

**Table 1:** Demographic and clinical information associated with pre-pandemic and pandemic cohorts, including maternal COVID-19 infection during pregnancy

	Pre-Pandemic (CTL)		Pandemic (RES)				Pandemic (RES)					
	total (*n* = 12)		total (*n* = 32)				COVID-19 negative (*n* = 17)		COVID-19 positive (*n* = 15)			
	mean	stdev	mean	stdev	*p*		mean	stdev	mean	stdev	*p*	
Infant gestational age (GA) at collection, in weeks	43.8	2.4	44.3	3.0	0.60		44.7	2.6	43.8	3.3	0.37	
Infant weight at delivery, in grams	3374	428	3381	558	0.97		3440	394	3315	710	0.54	
Infant head circumference at delivery, in cm	34.8	1.3	34	2	0.33		35	1	34	2	0.29	
Infant length at delivery, in cm	51.2	2.2	51	4	0.61		50	4	51	3	0.42	
Maternal age at birth, in years	34.3	4.1	32	6	0.33		35	4	29	6	***0.001***	
GA at COVID-19 diagnosis, in weeks									34	8		
	n	%	n	%	*z*	*p*	n	%	n	%	*z*	*p*
Smoking, maternal												
Yes	0	0	1	0	*n/a*	*n/a*	0	0	1	0	*n/a*	*n/a*
Self-reported race, infant												
White	8	67	11	34	1.93	0.05	10	59	1	7	3.10	***0.002***
Black	1	8	7	22	-1.04	0.30	2	12	5	33	-1.47	0.14
Asian	0	0	2	6	-0.89	0.37	2	12	0	0	1.37	0.17
Multiple/Other	2	17	3	9	0.68	0.50	2	12	1	7	0.49	0.62
Unknown	1	8	9	28	-1.39	0.16	1	6	8	53	-2.80	***0.002***
Self-reported ethnicity, infant												
Hispanic	1	8	10	31	-1.56	0.12	1	6	9	60	-3.30	***0.001***
Non-Hispanic	11	92	22	69	1.56	0.12	16	94	6	40	3.30	***0.001***
Infant sex												
Female	5	42	14	44	-0.12	0.90	5	29	9	60	-1.74	0.08
Male	7	58	18	56	0.12	0.9	12	71	6	40	1.74	0.08

Statistically significant *p*-values (≤ 0.05) are indicated in bold and italics. Collection methods for these data are detailed in the Methods section

### Neonatal buccal DNA extraction

Buccal swabs from the RESCUE cohort were collected for global DNA methylation analysis between June 2020 and February 2021 (i.e., prior to deployment of COVID-19 vaccines to the general population). All CTL swabs were collected prior to December 2019. ORAcollect buccal swabs (OG-175, DNA Genotek) were collected from neonates (average day of life 4.72 weeks ± 2.4), just prior to feeds and stored at 4˚C for batch processing. DNA was extracted with the PrepIT-L2P kit (DNA Genotek) and DNA was quantified using the Qubit Broad Range dsDNA assay kit and Qubit 4 fluorometer (Thermofisher Scientific). DNA was stored at -20˚C until ready for downstream applications.

### Genome-wide DNA methylation array

An input of 300 ng of DNA was bisulfite-converted using the DNA Methylation-Lightning kit (Zymo Research). After whole-genome amplification and enzymatic fragmentation, samples were hybridized to BeadChip arrays using the Infinium MethylationEPIC BeadChip kit according to the manufacturer’s protocol (Illumina). Intensity values at the over 850,000 methylation sites on the BeadChips were measured across the genome at single-nucleotide resolution using iScan (Illumina) or Nextseq 550 platform with BeadChip adapter (Illumina). Of note, all laboratory procedures from DNA extraction to DNA methylation array preparation and scanning were completed by one individual, in order to reduce variability between batches.

### Differential methylation analysis

We adopted the following pipeline for all further studies (Supplemental Fig. S1A). Quality filtration, functional normalization, and differential methylation analysis were performed on all arrays [35, 36]. Quality filtration was performed using *wateRmelon*, which removes probes if bead count of less than 3 in ≥ 5% of the samples [37]. Functional normalization of the data was executed using the *minfi* package with *ssnoob* background and dye correction [38]. Additional filtration using *minfi* excluded all probes that had a signal not significantly above background control probes (p-value > 0.05), all probes representing single nucleotide variants present in the general population with a minor allelic frequency > 5% (*dropLociWithSnps*), and cross-reactive probes (*maxprobes*) [39, 40].

### DNA methylation array pipeline

Because CTL samples were originally processed on the iScan platform, we included several replicates of CTL samples with each batch analysis of RES samples on the Nextseq platform to identify potential platform interference. None of the sample rows on either system failed during the scanning process and batch correction was used during bioinformatic analysis to account for the two distinct scanning platforms (Supplemental Fig. S1). To validate the analytic platform, we included all samples processed on these platforms in the same time-frame as those reported in this study; these include the 44 RES and CTL samples reported here, plus another 58 samples from other unrelated stress cohorts.

As seen in Supplemental Fig. S1, prior to any processing (865,859 probes), hierarchical clustering showed clustering of samples by platform and replicates did not cluster together (asterisks in Fig. S1B). After functional normalization, density plots of raw data (777,755 probes) were greatly improved (compare Supplemental Fig. S1C, E) but duplicates did not cluster together (Supplemental Fig. S1D). After batch correction, density plot is further improved (Supplemental Fig. S1G) and replicates cluster together (Supplemental Fig. S1F). We tested an alternative method for normalization, SWAN [41], but after SWAN and batch correction, while the replicates clustered (Supplemental Fig. S1H), the density plots appeared less ordered (Supplemental Fig. S1I).

### Statistical analysis

Beta-values were extracted, followed by batch correction and differential methylation analysis through linear regression models via *limma*. Replicate samples were run on each platform and in each batch to remove potential batch effects computationally. Our group previously reported significance thresholding using a commonly reported standard in the literature: results were considered statistically significant for differentially methylated probes (DMPs) with a false discovery rate (FDR) adjusted p-value < 0.05 and log_2_ fold-change ≥ 1 or ≤ -1 [40–43]. The networks and functional analyses were generated with QIAGEN IPA and Gene Ontology Enrichment Analysis GO with Enrichr [44, 45]; both tools used a statistical significance threshold of p ≤ 0.05. For pathway analysis (QIAGEN IPA), z-scores were calculated based off mean methylation differences at the multiple CpG sites (Illumina IDs) associated with annotated genes or pathways between CTL and RES cohorts (log_2_ fold-change). The z-score was calculated through the IPA software by assessing the overall log_2_ fold-change of all CpG sites associated with a specific pathway (i.e., predominantly negative fold-change differences are associated with hypomethylation in the RES compared to CTL and a negative z-score, where a majority positive fold-change indicates a positive z-score and hypermethylation in RES compared to control). A z-score of 0 means that the sites associated with a particular pathway included a mix of hyper- and hypomethylated sites (positive and negative fold-changes) that netted an overall score of zero. Graphs were made with ChAMP and ggplot2 in R [46, 47]. Comparisons of gene lists were performed on annotated probes only using Venny 2.1 [48].

## Results

We performed genome-wide DNA methylation analysis in two newborn cohorts, one born before the onset of the COVID-19 pandemic (CTL) and one recruited starting June 2020 (RES). Samples used in this study were age- and sex-matched (Table 1). Maternal age at delivery and height, weight, and length at delivery were not significantly different between the two cohorts (Table 1). Both cohorts were ethnically diverse and had a similar average maternal age; only one participant in this study indicated use of tobacco during pregnancy.

**Fig. 1:**
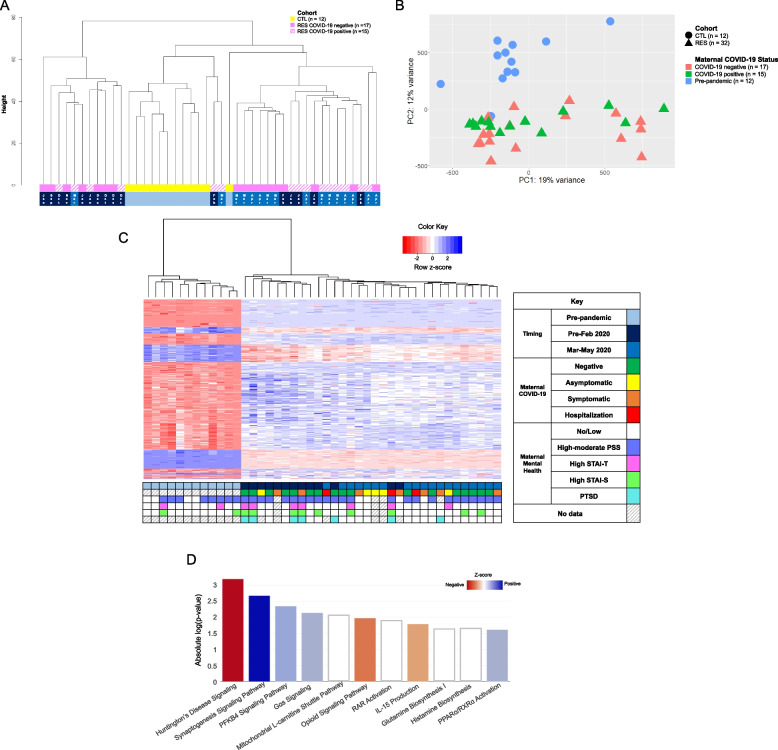
DNA methylation differences associated with newborns exposed to the COVID-19 pandemic in utero. **A** Dendrogram representation of unsupervised hierarchical clustering of normalized and batch-corrected beta values of pre-pandemic controls (CTL, yellow), RES pandemic COVID-19-negative pregnancies (solid pink), and RES COVID-19-positive pregnancies (hatched pink). Timing of approximate start of gestation is indicated at the bottom: light blue, entirety of pregnancy occurred prior to start of the pandemic (December 2019); dark blue; gestation that started September 2019-February 2020, prior to the onset of the COVID-19 pandemic; medium blue, gestation started between March and May 2020, after the US declared a national disaster. **B** Principal component analysis (PCA) of global DNA methylation differences of normalized and batch-corrected beta values between newborns exposed to the COVID-19 pandemic during pregnancy (RES, *n* = 32, triangles) and pre-pandemic healthy controls (CTL, *n* = 12, circles). Subcategorization by maternal COVID-19 infection status during pregnancy is denoted by color: coral indicates COVID-19 negative pregnancies (*n* = 17), green indicates COVID-19 positive pregnancies (*n* = 15), and blue indicates pre-pandemic pregnancies, unexposed to COVID-19 (*n* = 12). **C** Heatmap of unsupervised, hierarchical clustering of the differentially methylated probes identified between pre-pandemic CTL and RES pandemic newborns. Z-score scale represents transformed intensity values between differentially methylated probes with red being a negative z-score, white being a z-score of 0, and blue a positive z-score. The grid below the heatmap represents various clinical metrics, including timing of pregnancy (same color code as in 1A), COVID-19 infection status and severity of disease, and prenatal maternal mental health questionnaire data (STAI-S, STAI-T, PSS, PTSD), keyed in the legend to the right of the heatmap. Each column represents a sample, while rows represent specific probes that are differentially methylated between RES and CTL after performing linear regression analysis (FDR p-value ≤ 0.05, log_2_ fold-change threshold ≥ 1 or ≤ -1). **D** IPA analysis of annotated differentially methylated probes between CTL and RES cohort. Z-score scale was calculated from differential intensity values and red and blue shades indicate pathways with a negative or positive z-score, respectively. White indicates pathways with a z-score of 0 (genes in the pathway are differentially methylated, some positively, some negatively, resulting in a Z-score = 0). Absolute log(p-value) represents statistical significance for the identified pathway to be associated with the imported list of annotated probes

Unsupervised hierarchical clustering (Fig. 1a) of DMPs showed the pre-pandemic CTL cohort (in yellow) clustering away from the samples of the RES cohort (in pink). This was also visualized in principal component analysis (Fig. 1b) with clear separation and clustering of pre-pandemic newborns (circles) and newborns exposed to the COVID-19 pandemic during pregnancy (triangles). Linear regression analysis of CTL (*n* = 12) and RES cohort (*n* = 33) resulted in significant (FDR *p* ≤ 0.05) differential DNA methylation at 519 annotated sites (675 total probes) throughout the genome (Fig. 1c, Supplemental Table S2). Among the differentially methylated CpG sites, hypermethylation of *NR3C1*, a well-known epigenetic marker of prenatal stress, was observed in the RES cohort. Pathway analysis of these 519 differentially methylated sites suggests that increased methylation (positive z-score) may be associated with synaptogenesis signaling, PFKB4 signaling, Gs alpha signaling, and PPARɑ/RXRɑ activation, while decreased methylation (negative z-score) may be associated with neurodegenerative pathways (Huntington’s Disease), opioid signaling, and IL-15 production (Fig. 1d, Supplemental Table S3). Additional analysis of annotated DMPs using a distinct curated database (Supplemental Figure S3, Supplemental Table S4) confirmed association of DMPs with immune system pathways (*IL-5 and IL-13 production*) and nervous system developmental processes (*synapse structural plasticity*, *blood–brain-barrier permeability*).

Interestingly, while the CTL and RES cohorts showed striking differences in the methylation landscape genome-wide, the infection status of the mother during pregnancy did not seem to result in specific epigenetic signatures in the newborn (hashed vs. solid pink in Fig. 1a; green vs. orange triangles in Fig. 1b). There were no significant DMPs among the RES cohort between COVID-19 positive (*n* = 15) and negative (*n* = 17) pregnancies at the FDR p-value ≤ 0.05 and log_2_ fold-change ≥ 1 or ≤ -1 significance threshold, nor differences found in newborns exposed to asymptomatic, symptomatic, or hospitalized maternal COVID-19 infection (Fig. 1c green, yellow, orange, and red boxes).

**Fig. 2:**
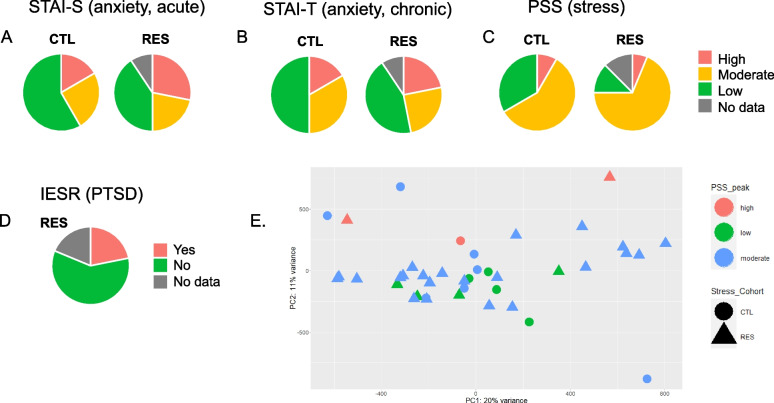
Answers to maternal mental health screening questionnaires. Maternal psychological distress was surveyed longitudinally throughout pregnancy and postnatally using the following surveys: **A**) STAI-S, **B**) STAI-T, **C**) PSS, and **D**) IESR (for RES only). Pie charts depict percentages of each cohort reporting high (red), moderate (orange), or low (green) stress or anxiety as well as presence (“yes”, red) or absence (“no”, green) of PTSD for the RES cohort only. **E** Example of PCA of DNA methylation intensity differences (non-significant) for maternal mental health (PSS) data. Maternal mental health data in tandem with DNA methylation data can be found PCAs can be found in Supplemental Figure S2

Maternal mental health metrics showed that overall, the RES cohort had a higher number of participants who scored with “high” state (STAI-S) and trait (STAI-T) anxiety and “moderate" or "high” stress (PSS) when compared to CTL (Fig. 2; data in Supplemental Table S1). Anxiety during pregnancy is estimated to affect 15–23% of women [49–51], which is a trend observable in the CTL cohort (~ 18% for both state and trait anxiety). While not significantly different from CTL, the RES cohort notably showed a trend toward a higher rate of both state and trait anxiety (30% and 21%, respectively) and stress (86% vs. 67% in CTL). Within RES, over a quarter of the women experienced PTSD symptoms at some point during pregnancy, even higher than the available report of 15% in the general population during the COVID-19 pandemic [52]. (The IESR questionnaire had not been used for the pre-pandemic CTL cohort). Using these additional mental health metrics (anxiety, stress, and PTSD), differential methylation analysis within the RES cohort (grouping high scores for STAI-S, STAI-T and high-moderate scores for PSS) did not identify significant methylation differences among these subcategories (Supplemental Figure S2; data in Supplemental Table S1). This can be further observed in the initial RES and CTL analysis, where there is no obvious clustering of DMPs associated with high stress, anxiety, or PTSD (Fig. 1c, fuchsia, lime, and aqua boxes).

Finally, due to the observed clustering among the RES cohort (Fig. 1a, pink) with one sub-cluster on either side of the (yellow) CTL cluster, we explored numerous metrics and found the timing of the pandemic during pregnancy to be linked with this pattern. "Early" pregnancies (gestation starting in late 2019-January 2020, before widespread knowledge of the pandemic, *n* = 13) and “late” pregnancies (conceived February-June 2020, *n* = 19) clearly segregated not only away from CTLs but also from each other (Fig. 1a). In the overall DNA methylation analysis between RES and CTL samples, there is distinct clustering of DMPs by timing (Fig. 1c, blue and navy boxes). Differential methylation analysis between RES newborns based on timing of pregnancy and CTLs identified 462 unique DMPs (632 total probes; Supplemental Table S5) associated with “early” 2020 pregnancies and 795 unique DMPs (1119 total probes; Supplemental Table S5) with “late” 2020 pregnancies, suggesting that the temporal aspect of the pandemic during pregnancy may have a unique impact on newborn DNA methylation. Further, we noted there was an overlap of 35.4% (331 annotated probes) in shared DMPs among all the resultant gene list from RES, “early” 2020, and “late” 2020 pandemic pregnancy newborns analysis against CTLs (Supplemental Figure S4). Finally, we performed a linear regression analysis between the early and late pandemic groups and found 324 sites of significant differential methylation, which further emphasizes the differences associated with timing of the pandemic on DNA methylation in these newborn cases (Supplemental Table 5).

## Discussion

In the present genome-wide DNA methylation study, significant differences were observed in otherwise healthy pregnancies during the pandemic when compared to pre-pandemic controls. With more than 500 annotated sites of differential methylation between these two groups, we observed significant global changes to the neonatal epigenomic landscape. Among the annotated sites of differential methylation, we noted methylation within the RES cohort at *NR3C1. NR3C1* is a glucocorticoid receptor gene, which has been associated with increased methylation in other studies of perinatal stress to newborns [53, 54], as well as trauma [55], psychological disorders, including major depression, post-traumatic stress disorder, anxiety, and personality disorders [56]. The findings presented here are consistent with existing literature that highlights the impact of prenatal stress on the newborn epigenome. Future studies will focus on validation of *NR3C1,* as well as the other differentially methylated sites presented in this study, as biomarkers of perinatal stress and aim to assess any potential associations between neonatal epigenomics with long-term neurodevelopmental outcomes.

Top pathways associated with differential methylation in the RES cohort included *IL-15 production*. Maternal stress during pregnancy has been strongly linked to increased inflammation and risk for future neuropsychiatric outcomes in offspring [57]. IL-15 is a proinflammatory cytokine that is critical for healthy maternal and fetal outcomes in pregnancy [58], and upregulation of IL-15 during pregnancy has been correlated with adverse outcomes of pregnancy, such as preeclampsia and gestational diabetes mellitus [59, 60], and with neonatal neurodevelopmental delay [61]. While IL-15 production is a key biological response to viral infection, this pathway was more methylated regardless of viral infection status in the mother. Its potential downregulation in newborns of the pandemic may function as a protective mechanism against negative developmental brain outcomes.

The neurodegenerative disease pathway, *Huntington’s disease signaling* and *synaptogenesis signaling* were also top terms significantly associated with differential methylation in pandemic pregnancy newborns. Impaired neurodevelopmental outcomes are a strongly correlated with fetal stress and often associated with reduced infant brain plasticity and dysregulated developmental processes, such as synaptogenesis, neuronal migration, and myelination [62, 63]. Mitochondrial dysfunction, inflammation, and oxidative stress are key players in neurodegeneration and neurodegenerative diseases, such as Huntington’s disease [64, 65], that also have been shown to be triggered by stress during pregnancy and perhaps lead to impaired neurodevelopment in newborns [66–68]. Together, this combination of pathways may provide insight into the mechanisms that underpin the impact of fetal stressors during pregnancy and long-term impaired neurodevelopmental outcomes that follow.

Another top hypomethylated pathway in the RES cohort was the PFKB4 pathway. The PFKB4 is both a regulator of glycolysis and a regulator of progression of ectodermal patterning toward specific fates including neural plate and neural crest during embryonic development, independently of glycolysis [69]. It also promotes tumor growth and metastasis [70], and whether this will have an impact on developmental outcomes remains to be seen. It will be critical to document longitudinal developmental outcomes in both CTL and RES cohort to test these mechanistic hypotheses.

As there were no significant differences in DNA methylation associated with maternal COVID-19 infection in this analysis, it may be inferred that the changes occurring to the neonatal are epigenome are likely a direct result of the COVID-19 pandemic environment, rather than maternal COVID-19 infection status during pregnancy. This is congruent with the literature, which suggests that there is likely only rare instances of vertical intrauterine transmission of the SARS-CoV-2 virus from infected mothers to their fetuses [53, 71].

Finally, after noticing that two distinct clusters emerged among the pandemic RES cohort, we subset the DNA methylation analysis to examine whether the onset ("early" group) and continuation ("late" group) of the COVID-19 pandemic may have resulted in different and significant changes in this cohort. Here, we noted over 462 unique annotated sites of differential methylation between the “early” 2020 pandemic group and CTL and 795 annotated sites between the “late” 2020 pandemic group and CTL (Supplemental Table S5). The “early” group included women who were in their first trimester immediately before to the onset of the COVID-19 pandemic (between September 2019 and January 2019), while the late 2020 group included women who were in their first trimester between February 2020 and May 2020 and thus directly impacted by the COVID-19 pandemic for the entirety of their pregnancy. When looking at all groups combined, we noted 77 annotated sites unique to the “early” pandemic RES cohort and 346 unique to the “late” pandemic RES cohort, with an overlap of 331 annotated sites between early, late, and all RES pandemic pregnancy cohort newborns (Supplemental Figure S4). To further emphasize the differences associated with the timing of pandemic exposure, we conducted an additional differential methylation analysis between the early and late pandemic groups, which resulted in a difference of 324 sites (Supplemental Table 5). Published reports suggest that the timing of stress during pregnancy and length of exposure may play an important role in the ultimate postnatal outcomes of a child and, as such, could play a role the differences in DNA methylation within the RES cohort [72, 73]. For example, a 2018 study focused on “military stress” during pregnancy subset their analysis between “new onset” and “chronic” stress exposure, as well as the trimester that the stress occurred [72]. While the exact nature of these differences has yet to be fully described, the present data suggest a distinct and significant impact that the timing and type of distress induced by the COVID-19 pandemic during pregnancy had on the newborn epigenetic landscape. Additionally, future studies may benefit from considering the concept of chronoepigenetics, or study of epigenetic changes over time, to better understand the impact of insults and stressors during vulnerable periods of development and how they may cause critical changes to the fetal and neonatal epigenome, as well as considering additional factors such regionally diverse datasets that may have been differentially impacted by COVID-19 pandemic policies and guidelines [73].

A few limitations impact this study: availability of COVID-19 testing, missing or non-compliance with completing self-reported psychosocial surveys, and cohort size. Maternal COVID-19 infection status was determined by positive testing noted in the medical records, as well self-reported testing by participants of the RES study. Participants among the RES cohort without known exposures or asymptomatic COVID-19 infections may not have been accurately captured. Also, availability of testing evolved during enrollment for this study. Maternal mental health measures between RES and CTL did not reach statistical significance, however we were likely underpowered given the sample size of the pre-pandemic cohort (*n* = 12). The maternal mental health surveys (STAI-S, STAI-T, PSS, and IESR) used may not fully capture the unprecedented or dynamic nature of the type of distress that occurred throughout the COVID-19 pandemic. Restriction in access to hospital settings for healthy research volunteers imposed during the pandemic limited study visits for the CTL cohort and missing data, as well as recruitment bias specific to the Washington DC-Metropolitan area, may skew the mental health data acquired in this study [74]. Of note, while there was no difference in average gestational ages at time of collection between the RES and CTL cohorts, buccal swabs were collected within a 2–4 week period after birth (Table 1). Therefore, the data presented here may not be representative of the newborn genome immediately after birth, nor does it represent all COVID-19 pandemic or otherwise healthy pregnancies [74]. Our group has previously reported that the overall paucity and disparity of publicly available data during the onset and continuation of the COVID-19 pandemic has been a pervasive issue and ultimately hampers medical, research, and public health policy decisions [75, 76]. The present study included a total of 44 participants, which is a small sample size for global DNA methylation array studies. To compensate for this, we employed stringent differential methylation criteria (i.e., FDR *p*-value < 0.05, effect size cutoff of log_2_ fold-change cutoff of greater than 1 or less than -1). While this improves the statistical rigor of the subsequent differential methylation analysis, we once again emphasize that a larger, well-powered sample population is preferable for a study of this nature. We also acknowledge that while stringent filtering criteria, statistical thresholds, and batch correction was employed in our bioinformatic analysis, potential batch effects, false positives, and confounding factors (time of collection, early infant environmental exposures, etc.) may still exist and impact the data presented here [77]. The breadth of the changes, however, warranted rapid reporting even with these limitations, as the data presented in this study could be of critical importance from a research and public health perspective.

## Conclusions

In this study, we found a striking alteration of the neonatal epigenome coincident with the COVID-19 pandemic, but not maternal COVID-19 infection, a factor that must be considered in future DNA methylation studies comparing pre-pandemic data. While there was not significant correlation between maternal mental health survey data (stress, anxiety, depression), fetuses exposed to the onset and continuation of COVID-19 pandemic throughout 2020 had over 500 sites of significant differential DNA methylation, including *NR3C1* and other genes associated with pathways involved in neurodevelopment and immune system response. Of note, there also appears to be a temporal aspect of the pandemic on the neonatal epigenome with divergent signatures appearing among infants exposed to just the onset of the pandemic in early 2020 and those exposed to the continuation of the pandemic throughout mid-late 2020. Overall, the widespread differences seen in DNA methylation between pre-pandemic and pandemic-exposed newborns provides critical insight into future development of these cohorts, which should be carefully documented longitudinally.

### Supplementary Information

**Additional file 1: Supplemental Figure S1.** Methodology of DNA methylation analysis using 850k EPIC array.

**Additional file 2:** **Supplemental Figure S2.** Principal Component Analysis of differentially methylated sites for each maternal psychological status assessment instrument: A) STAIS, B) STAI-T, D) PSS, and D) IESR (for RES only). A-C) triangles represent RES while circles represent CTL; all scores (STAI-S, STAI-T, PSS) are broken down in the following: “low” is green, “moderate” is blue, and “high” is red. D) IES-R scores are dichotomized into “no PTSD” (red) and “PTSD” (teal).There were no sites of differential methylation associated with any maternal mental health metric at a statistical threshold of FDR p < 0.05 and log2 fold-change of 1> or <-1.

**Additional file 3: Supplemental Figure S3.** GO enrichment analysis of annotated DMPs between RES and CTL cohorts.

**Additional file 4: Supplemental Figure S4. **Venn diagram of unique and overlapping differentially methylated genes between early and late 2020 pandemic RES subcohorts.

**Additional file 5: Supplementary Table S1.** Maternal psychological stress surveyed throughout pregnancy in pre-pandemic and pandemic cohorts.

**Additional file 6: Supplementary Table S2.** Differentially methylated probes and annotated genes between pre-pandemic and pandemic newborns.

**Additional file 7: Supplementary Table S3. **Pathway analysis of differentially methylated genes associated with pandemic newborns.

**Additional file 8: Supplementary Table S4. **Gene Ontology Enrichment analysis of differentially methylated genes associated with pandemic newborns.

**Additional file 9: Supplementary Table 5. **Sites of differential methylation between early and late 2020 pandemic newborns.

## Data Availability

The datasets generated and analyzed in the current study are pending approval but will be available in the Gene Expression Omnibus (GEO) repository. Until the public release date, 2023–12-31, please use this temporary link to access the data used in this study (GSE229463). GEO data access token: azadmymilhcvlcb.

## Abbreviations

COVID-19: Coronavirus Disease 2019
RESCUE, RES: Reducing Elevated Stress from COVID-19 Exposure
CTL: Control
GA: Gestational age
STAI: State-Trait Anxiety Inventory
PSS: Perceived Stress Scale
IESR: Impact of Event Scale Revised
PTSD: Post-traumatic stress disorder
DMP: Differentially methylated probe
