# Supplementary material for: Genome-wide neonatal epigenetic changes associated with maternal exposure to the COVID-19 pandemic
Source: BMC Med Genomics. 2023 Oct 30;16:268. doi: 10.1186/s12920-023-01707-4 (PMC10614377; doi:10.1186/s12920-023-01707-4)
Supplement: Supplementary file 1 — Additional file 1: Supplemental Figure S1. Methodology of DNA methylation analysis using 850k EPIC array. [file 12920_2023_1707_MOESM1_ESM.pptx]

## Slide 1
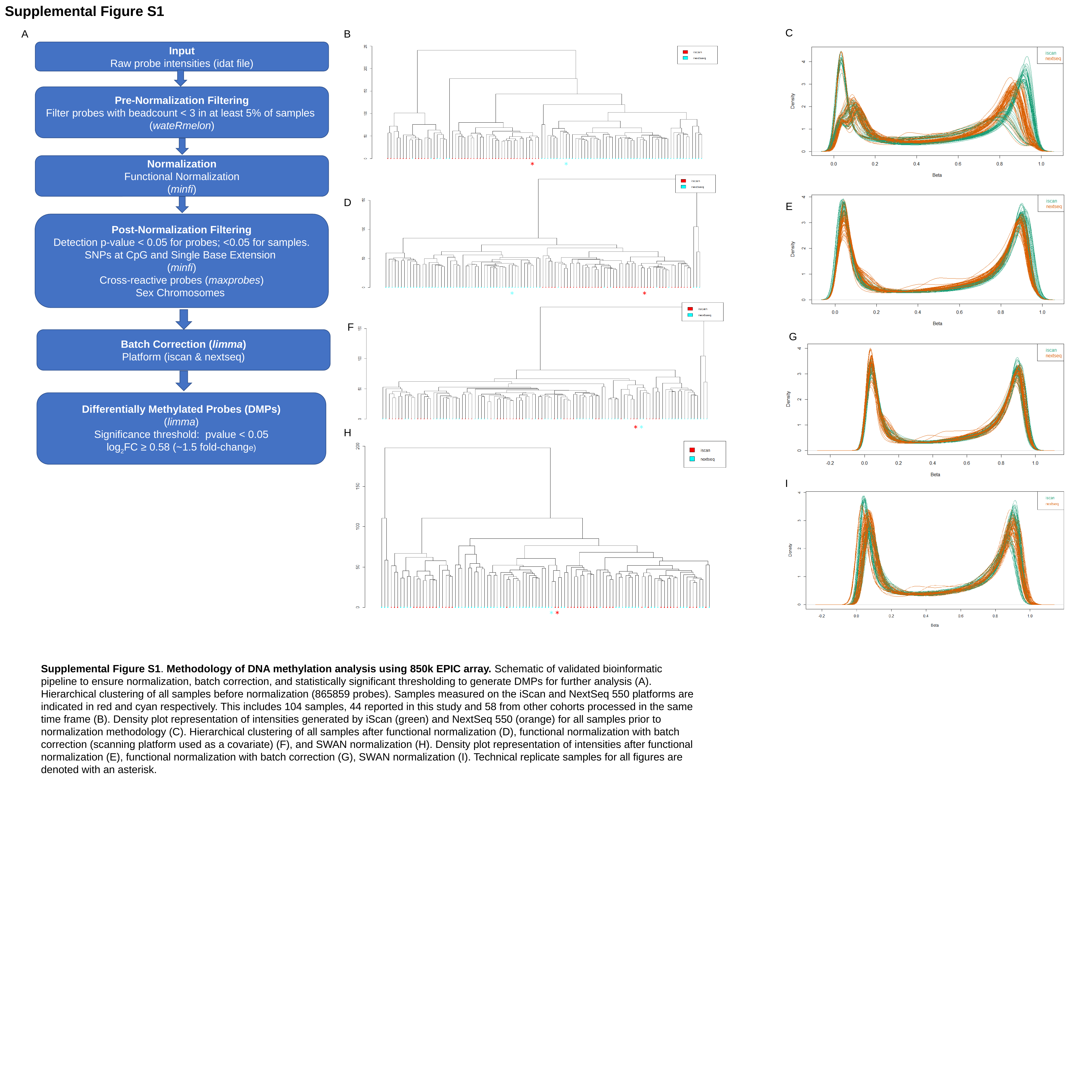

Supplemental Figure S1
C
A
B
Input
Raw probe intensities (idat file)
Pre-Normalization Filtering
Filter probes with beadcount < 3 in at least 5% of samples
(wateRmelon)
Normalization
Functional Normalization
(minfi)
Post-Normalization Filtering
Detection p-value < 0.05 for probes; <0.05 for samples.
SNPs at CpG and Single Base Extension
(minfi)
Cross-reactive probes (maxprobes)
Sex Chromosomes
Batch Correction (limma)
Platform (iscan & nextseq)
Differentially Methylated Probes (DMPs)
(limma)
Significance threshold: pvalue < 0.05
log2FC ≥ 0.58 (~1.5 fold-change)
*
*
*
*
D
E
F
G
**
H
I
**
Supplemental Figure S1. Methodology of DNA methylation analysis using 850k EPIC array. Schematic of validated bioinformatic pipeline to ensure normalization, batch correction, and statistically significant thresholding to generate DMPs for further analysis (A). Hierarchical clustering of all samples before normalization (865859 probes). Samples measured on the iScan and NextSeq 550 platforms are indicated in red and cyan respectively. This includes 104 samples, 44 reported in this study and 58 from other cohorts processed in the same time frame (B). Density plot representation of intensities generated by iScan (green) and NextSeq 550 (orange) for all samples prior to normalization methodology (C). Hierarchical clustering of all samples after functional normalization (D), functional normalization with batch correction (scanning platform used as a covariate) (F), and SWAN normalization (H). Density plot representation of intensities after functional normalization (E), functional normalization with batch correction (G), SWAN normalization (I). Technical replicate samples for all figures are denoted with an asterisk.
