# Supplementary material for: Genome-wide neonatal epigenetic changes associated with maternal exposure to the COVID-19 pandemic
Source: BMC Med Genomics. 2023 Oct 30;16:268. doi: 10.1186/s12920-023-01707-4 (PMC10614377; doi:10.1186/s12920-023-01707-4)
Supplement: Supplementary file 2 — Additional file 2: Supplemental Figure S2. Principal Component Analysis of differentially methylated sites for each maternal psychological status assessment instrument: A) STAIS, B) STAI-T, D) PSS, and D) IESR (for RES only). A-C) triangles represent RES while circles represent CTL; all scores (STAI-S, STAI-T, PSS) are broken down in the following: “low” is green, “moderate” is blue, and “high” is red. D) IES-R scores are dichotomized into “no PTSD” (red) and “PTSD” (teal).There were no sites of differential methylation associated with any maternal mental health metric at a statistical threshold of FDR p < 0.05 and log2 fold-change of 1> or <-1. [file 12920_2023_1707_MOESM2_ESM.pptx]

## Slide 1
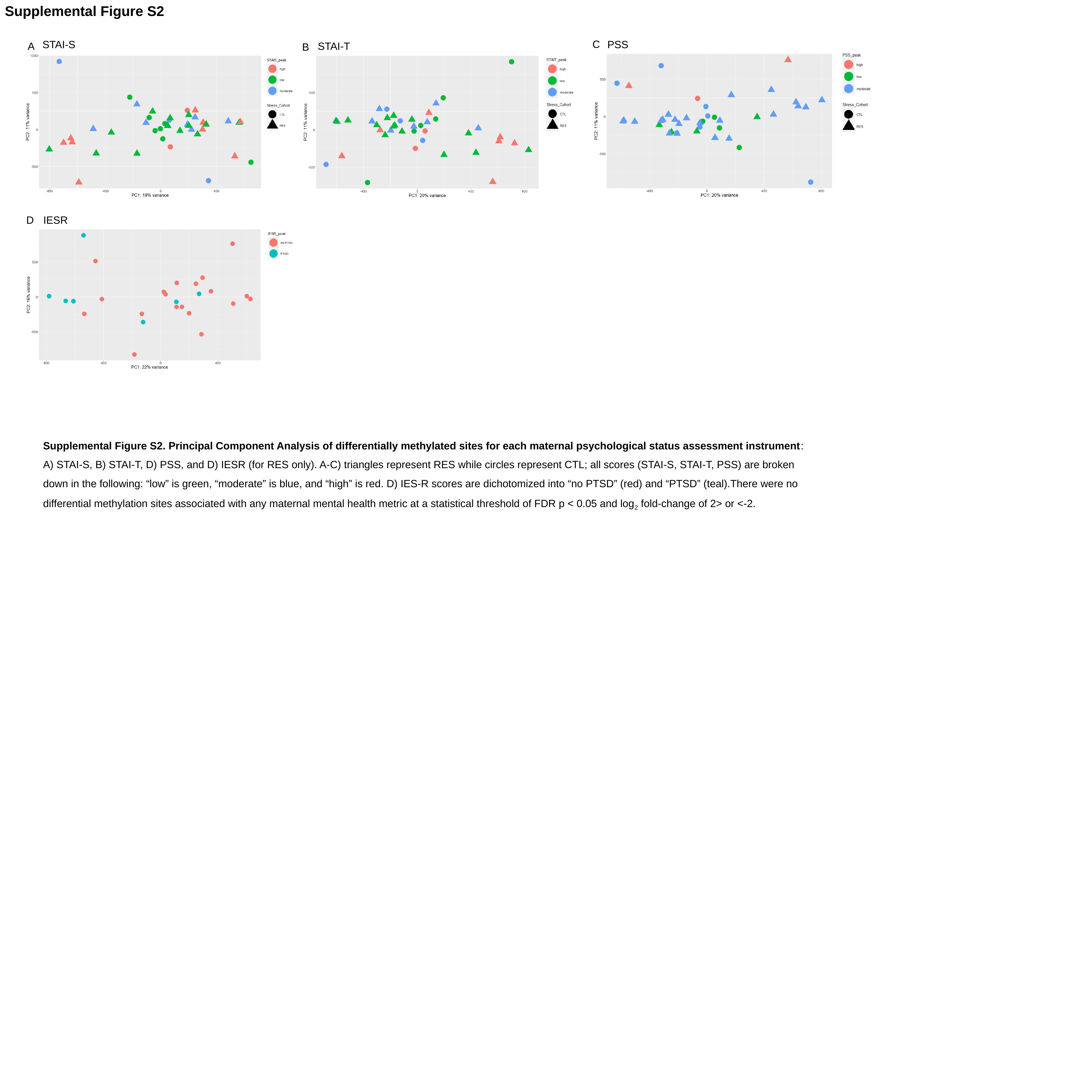

Supplemental Figure S2
C
STAI-S
PSS
A
STAI-T
B
D
IESR
Supplemental Figure S2. Principal Component Analysis of differentially methylated sites for each maternal psychological status assessment instrument: A) STAI-S, B) STAI-T, D) PSS, and D) IESR (for RES only). A-C) triangles represent RES while circles represent CTL; all scores (STAI-S, STAI-T, PSS) are broken down in the following: “low” is green, “moderate” is blue, and “high” is red. D) IES-R scores are dichotomized into “no PTSD” (red) and “PTSD” (teal).There were no differential methylation sites associated with any maternal mental health metric at a statistical threshold of FDR p < 0.05 and log2 fold-change of 2> or <-2.
