# Supplementary material for: Genome-wide neonatal epigenetic changes associated with maternal exposure to the COVID-19 pandemic
Source: BMC Med Genomics. 2023 Oct 30;16:268. doi: 10.1186/s12920-023-01707-4 (PMC10614377; doi:10.1186/s12920-023-01707-4)
Supplement: Supplementary file 3 — Additional file 3: Supplemental Figure S3. GO enrichment analysis of annotated DMPs between RES and CTL cohorts. [file 12920_2023_1707_MOESM3_ESM.pptx]

## Slide 1
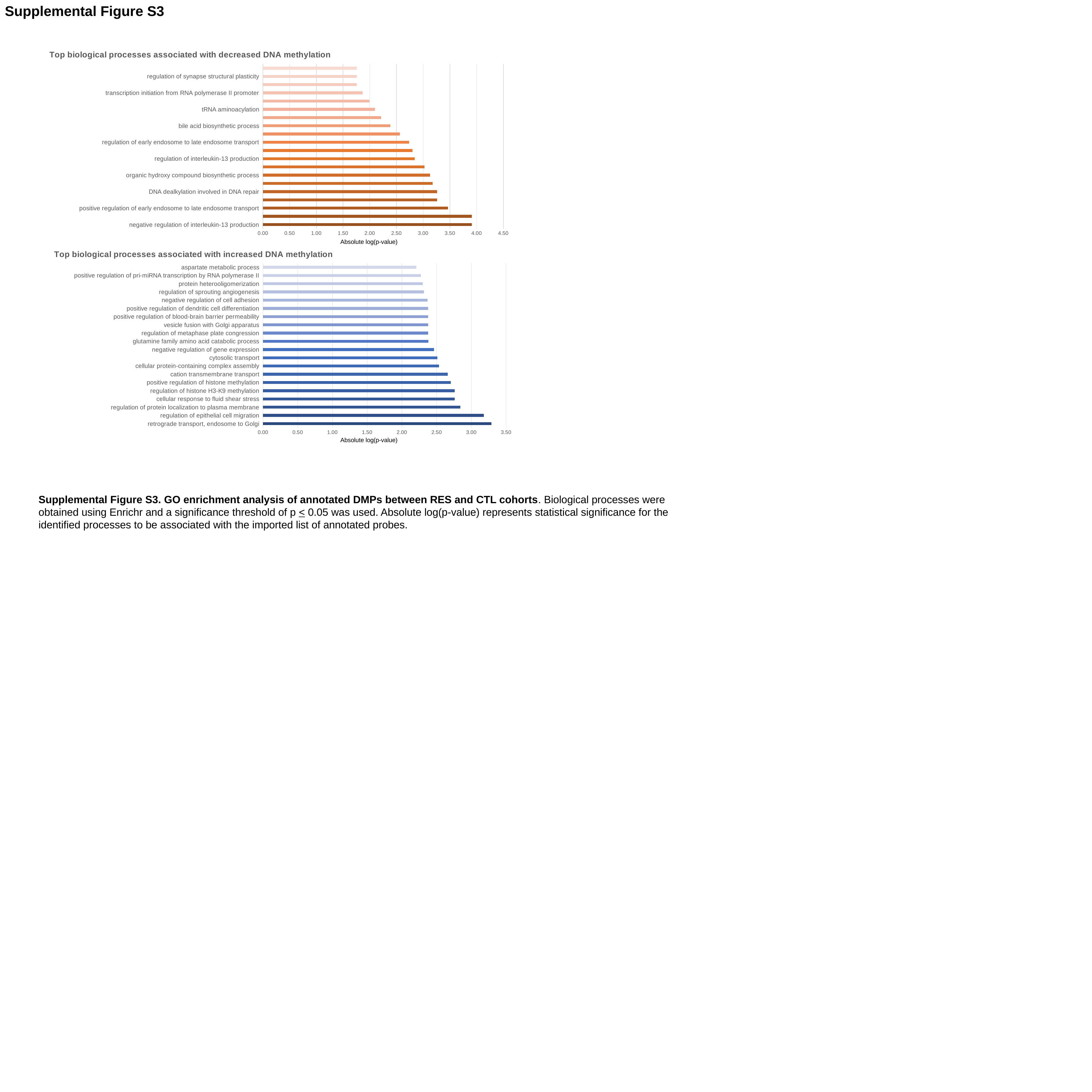

Supplemental Figure S3
### Chart: Top biological processes associated with decreased DNA methylation
| Category | absolute_logpval |
|---|---|
| negative regulation of interleukin-13 production | 3.908698401370608 |
| negative regulation of interleukin-5 production | 3.908698401370608 |
| positive regulation of early endosome to late endosome transport | 3.464534205705133 |
| DNA dealkylation | 3.260475235867698 |
| DNA dealkylation involved in DNA repair | 3.260475235867698 |
| positive regulation of cytoplasmic transport | 3.1743225810544615 |
| organic hydroxy compound biosynthetic process | 3.1275402022462697 |
| regulation of interleukin-5 production | 3.024585147388574 |
| regulation of interleukin-13 production | 2.840488664336616 |
| steroid biosynthetic process | 2.7960265941217544 |
| regulation of early endosome to late endosome transport | 2.7369708067182996 |
| ubiquitin-dependent protein catabolic process via the multivesicular body sorting pathway | 2.5620321103929196 |
| bile acid biosynthetic process | 2.385308867590002 |
| bile acid metabolic process | 2.2139401833185004 |
| tRNA aminoacylation | 2.094576982873731 |
| tRNA aminoacylation for protein translation | 1.9907964214460263 |
| transcription initiation from RNA polymerase II promoter | 1.867480918954505 |
| regulation of receptor catabolic process | 1.7538454850336742 |
| regulation of synapse structural plasticity | 1.7538454850336742 |
| regulation of adherens junction organization | 1.7538454850336742 |Absolute log(p-value)
### Chart: Top biological processes associated with increased DNA methylation
| Category | absolute_logpval |
|---|---|
| retrograde transport, endosome to Golgi | 3.286528584556493 |
| regulation of epithelial cell migration | 3.1790921151374194 |
| regulation of protein localization to plasma membrane | 2.84234438906751 |
| cellular response to fluid shear stress | 2.758295403985434 |
| regulation of histone H3-K9 methylation | 2.758295403985434 |
| positive regulation of histone methylation | 2.7055538789234195 |
| cation transmembrane transport | 2.6605102876724636 |
| cellular protein-containing complex assembly | 2.5336056174416015 |
| cytosolic transport | 2.511083783195029 |
| negative regulation of gene expression | 2.463780918243618 |
| glutamine family amino acid catabolic process | 2.379546356254065 |
| regulation of metaphase plate congression | 2.3768617236506207 |
| vesicle fusion with Golgi apparatus | 2.3768617236506207 |
| positive regulation of blood-brain barrier permeability | 2.3768617236506207 |
| positive regulation of dendritic cell differentiation | 2.3768617236506207 |
| negative regulation of cell adhesion | 2.3706856149117836 |
| regulation of sprouting angiogenesis | 2.31826466252133 |
| protein heterooligomerization | 2.301961441100408 |
| positive regulation of pri-miRNA transcription by RNA polymerase II | 2.271029158625111 |
| aspartate metabolic process | 2.2068175369065184 |Absolute log(p-value)
Supplemental Figure S3. GO enrichment analysis of annotated DMPs between RES and CTL cohorts. Biological processes were obtained using Enrichr and a significance threshold of p < 0.05 was used. Absolute log(p-value) represents statistical significance for the identified processes to be associated with the imported list of annotated probes.
