# Supplementary material for: Genome-wide neonatal epigenetic changes associated with maternal exposure to the COVID-19 pandemic
Source: BMC Med Genomics. 2023 Oct 30;16:268. doi: 10.1186/s12920-023-01707-4 (PMC10614377; doi:10.1186/s12920-023-01707-4)
Supplement: Supplementary file 4 — Additional file 4: Supplemental Figure S4. Venn diagram of unique and overlapping differentially methylated genes between early and late 2020 pandemic RES subcohorts. [file 12920_2023_1707_MOESM4_ESM.pptx]

## Slide 1
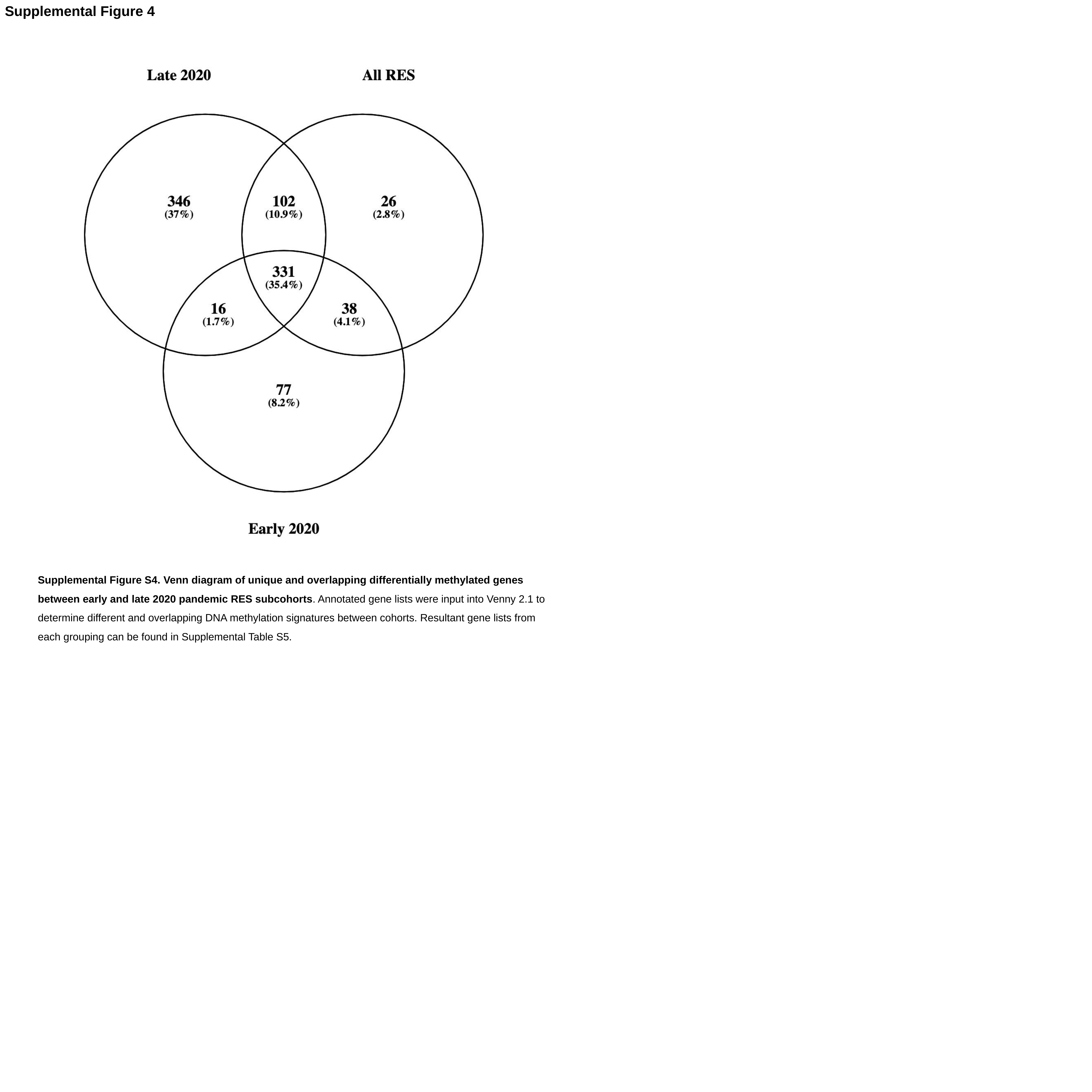

Supplemental Figure 4
Supplemental Figure S4. Venn diagram of unique and overlapping differentially methylated genes between early and late 2020 pandemic RES subcohorts. Annotated gene lists were input into Venny 2.1 to determine different and overlapping DNA methylation signatures between cohorts. Resultant gene lists from each grouping can be found in Supplemental Table S5.
